# Mac‐2 Binding Protein Glycosylation Isomer (M2BPGi)–Based Risk Stratification Refined by Tumor Metabolic Activity in Hepatocellular Carcinoma

**DOI:** 10.1002/ags3.70272

**Published:** 2026-09-01

**Authors:** Kojiro Shirabe, Junya Mita, Shinji Itoh, Takeo Toshima, Yoshiyuki Kitamura, Norifumi Iseda, Takuro Isoda, Kousei Ishigami, Tomoharu Yoshizumi

**Affiliations:** ^1^ Department of Surgery and Science, Graduate School of Medical Sciences Kyushu University Fukuoka Japan; ^2^ Department of Clinical Radiology, Graduate School of Medical Sciences Kyushu University Fukuoka Japan

**Keywords:** hepatic fibrosis, hepatic resection, hepatocellular carcinoma, Mac‐2 binding protein glycosylation isomer, tumor metabolism

## Abstract

**Aim:**

To evaluate the prognostic significance of preoperative Mac‐2 binding protein glycosylation isomer (M2BPGi) and determine whether tumor metabolic activity provides additional risk stratification after hepatic resection for hepatocellular carcinoma (HCC).

**Methods:**

We retrospectively analyzed 291 patients with HCC who underwent hepatic resection at a single Japanese institution between 2015 and 2023 and had available preoperative M2BPGi and 18F‐fluorodeoxyglucose positron emission tomography/computed tomography data. Associations with clinicopathological features, recurrence‐free survival (RFS), and overall survival (OS) were evaluated.

**Results:**

High M2BPGi was associated with impaired liver function and F3–F4 fibrosis but not with tumor‐related features or tumor metabolic activity. In contrast, high tumor metabolic activity was associated with higher des‐*γ*‐carboxy prothrombin (DCP) levels, larger tumors, poor differentiation, microscopic vascular invasion, and intrahepatic metastasis. High M2BPGi and high tumor metabolic activity independently predicted worse RFS (hazard ratio [HR], 1.61; *p* = 0.0105 and HR, 1.94; *p* = 0.0023, respectively) and OS (HR, 2.03; *p* = 0.0042 and HR, 1.93; *p* = 0.0199, respectively). The dual‐high group had the poorest RFS and OS (both *p* < 0.0001). In public data analysis, M2BPGi‐related fibrosis and glycolysis‐related signatures were positively correlated with a mechanistic target of rapamycin complex 1 (mTORC1)‐related anabolic signature (*R* = 0.439 and *R* = 0.670, respectively; *p* < 0.0001), and a high mTORC1‐related signature was associated with worse OS (*p* = 0.0074).

**Conclusions:**

Preoperative M2BPGi identifies fibrosis‐related risk after HCC resection, whereas tumor metabolic activity further distinguishes patients with particularly poor outcomes.

## Graphical abstract

M2BPGi reflects background liver fibrosis and hepatic functional impairment. PET SUVmax reflects tumor metabolic aggressiveness. Their combination identifies a dual‐high subgroup with the poorest recurrence‐free and overall survival.

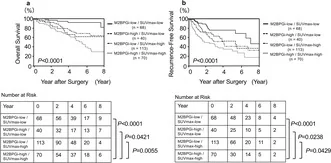

**Abbreviations**
^18^F‐FDG: ^18^F‐fluorodeoxyglucose
AFP: alpha‐fetoprotein
ALBI: albumin–bilirubin
AST: aspartate aminotransferase
AUC: area under the curve
CI: confidence interval
CT: computed tomography
DCP: des‐*γ*‐carboxy prothrombin
GEPIA3: Gene Expression Profiling Interactive Analysis 3
HCC: hepatocellular carcinoma
HR: hazard ratio
IQR: interquartile range
M2BPGi: Mac‐2 binding protein glycosylation isomer
mALBI: modified albumin–bilirubin
MSigDB: Molecular Signatures Database
mTORC1: mechanistic target of rapamycin complex 1
OS: overall survival
PET: positron emission tomography
RFS: recurrence‐free survival
ROC: receiver operating characteristic
SUVmax: maximum standardized uptake value
TCGA‐LIHC: The Cancer Genome Atlas Liver Hepatocellular Carcinoma project
TPM: transcripts per million

## 1 Introduction

Hepatocellular carcinoma (HCC) often recurs even after curative hepatic resection [1, 2, 3]. Postoperative outcomes are influenced not only by tumor aggressiveness but also by the condition of the background liver [4, 5]. Therefore, both factors should be considered when estimating prognosis after resection.

Mac‐2 binding protein glycosylation isomer (M2BPGi) is a serum marker of liver fibrosis and has been associated with HCC development and recurrence after hepatic resection [5, 6, 7]. In contrast to conventional liver function parameters, M2BPGi primarily reflects the fibrotic condition of the background liver and may therefore provide additional prognostic information preoperatively. However, prognosis after HCC resection also depends on the biological aggressiveness of the tumor, which cannot be fully captured by a fibrosis‐related marker alone.

Because M2BPGi reflects the background liver, we examined tumor metabolic activity as a complementary prognostic factor. Tumor glucose metabolism was assessed using the maximum standardized uptake value (SUVmax) on ^18^F‐fluorodeoxyglucose positron emission tomography/computed tomography (^18^F‐FDG PET/CT), a measure associated with poor differentiation, vascular invasion, and unfavorable outcomes in HCC [8, 9]. Thus, M2BPGi and SUVmax may represent two distinct but complementary components of postoperative risk: the condition of the background liver and the metabolic aggressiveness of the tumor. However, the prognostic value of combining these markers has not been fully examined.

In this study, we evaluated the clinicopathological and prognostic significance of preoperative M2BPGi and examined whether its combination with tumor metabolic activity could improve postoperative risk stratification after hepatic resection for HCC.

## 2 Methods

### 2.1 Patients

We retrospectively reviewed 291 patients with HCC who underwent hepatic resection at the Department of Surgery and Science, Kyushu University Hospital, between April 2015 and December 2023. The indications for surgery and the operative procedures used at our institution have been described previously [10]. Among these cases, 71 (24.4%) had received treatment for HCC before the index hepatectomy, including hepatic resection, transarterial chemoembolization, radiofrequency ablation, or chemotherapy; some had received more than one treatment type. After discharge, patients were followed every 1–3 months in the outpatient clinic. Dynamic computed tomography (CT) was generally performed every 3 months, and magnetic resonance imaging was added when recurrence was suspected. Clinical and follow‐up data were obtained from electronic medical records. The study protocol was approved by the Ethics Committee of Kyushu University (approval code: 23339).

### 2.2 Measurement of M2BPGi

Serum M2BPGi levels were obtained from routine laboratory tests performed before hepatic resection. When more than one preoperative measurement was available, the value closest to the date of surgery was used. The cutoff value for M2BPGi was determined using conventional receiver operating characteristic (ROC) curve analysis based on vital status during follow‐up. No prespecified landmark time point was used. The optimal cutoff value was defined as the value that maximized the Youden index (sensitivity + specificity − 1).

### 2.3 ^18^F‐FDG PET/CT Imaging and SUVmax Assessment

After fasting for at least 4 h, each patient received 185 MBq of ^18^F‐FDG intravenously. PET/CT imaging was started 60 min after injection and extended from the mid‐thigh to the skull vertex. Examinations were conducted using either a Discovery STE scanner (GE Medical Systems, Milwaukee, WI, USA) or a Biograph mCT scanner (Siemens Medical Solutions, Erlangen, Germany). Emission data were acquired in three‐dimensional mode. The acquisition time was 3 min per bed position for the Discovery STE and 2 min per bed position for the Biograph mCT. Images obtained with the Discovery STE were reconstructed using the ordered‐subset expectation–maximization algorithm (VUE Point Plus) with two iterations and 28 subsets. Images obtained with the Biograph mCT were reconstructed using the iterative TrueX algorithm with time‐of‐flight imaging (Ultra HD‐PET), with two iterations and 21 subsets. The TrueX algorithm incorporates point‐spread‐function correction. Spatial resolution, expressed as full width at half maximum, was 5.2 mm for the Discovery STE and 4.4 mm for the Biograph mCT. Low‐dose CT was performed before PET for attenuation correction and anatomical localization. The Discovery STE was equipped with a 16‐slice CT scanner operated at 120 kV and 30–250 mA, whereas the Biograph mCT used a 32‐slice CT scanner operated at 120 kV with angular and longitudinal dose modulation using CARE Dose 4D. CT images were reconstructed by filtered back‐projection into a 512 × 512 matrix with a slice thickness of 5 mm, corresponding to the PET slice interval. Tumoral ^18^F‐FDG uptake was assessed using SUVmax, which was calculated on the dedicated workstation for each scanner. The cutoff value for SUVmax was determined using conventional ROC curve analysis based on vital status during follow‐up. No prespecified landmark time point was used. The optimal cutoff value was defined as the value that maximized the Youden index (sensitivity + specificity − 1).

### 2.4 Public Database Analysis

Exploratory transcriptomic analysis was performed in Gene Expression Profiling Interactive Analysis 3 (GEPIA3) using the tumor cohort from The Cancer Genome Atlas Liver Hepatocellular Carcinoma project (TCGA‐LIHC). In GEPIA3, each gene‐set score was derived from the first principal component of the expression levels of the included genes after log_2_(TPM + 1) transformation. Three non‐overlapping gene sets were defined before the survival analysis based on published biological evidence and pathway annotations rather than on their prognostic associations in TCGA‐LIHC.

The mechanistic target of rapamycin complex 1 (mTORC1)‐related anabolic signature comprised ASNS, MTHFD2, PSAT1, SLC7A5, and RRM2. All five genes are included in the Molecular Signatures Database (MSigDB) HALLMARK_MTORC1_SIGNALING gene set. These genes were selected as a compact panel representing complementary components of mTORC1‐associated anabolic metabolism, including amino acid synthesis and transport, serine and one‐carbon metabolism, and nucleotide synthesis [11]. The M2BPGi‐related fibrosis signature comprised LGALS3BP, ACTA2, COL1A1, and COL3A1. LGALS3BP was included because it encodes M2BP, the protein backbone of M2BPGi [12]. ACTA2 was selected as a marker of activated hepatic stellate cells. In human fibrotic liver, M2BP and ACTA2 transcripts have been shown to colocalize in hepatic stellate cells, and hepatic M2BP transcript expression was positively associated with serum M2BPGi levels [13]. COL1A1 and COL3A1 were included to represent collagen‐rich extracellular matrix production associated with hepatic stellate cell activation and liver fibrosis [14]. The glycolysis‐related signature comprised SLC2A1, HK2, PFKP, PKM, and LDHA, representing sequential steps in glycolytic metabolism from glucose uptake and phosphorylation to pyruvate and lactate production. SLC2A1, which encodes GLUT1, was included to reflect glucose uptake relevant to ^18^F‐FDG accumulation [15]. HK2, PFKP, PKM, and LDHA are included in the MSigDB HALLMARK_GLYCOLYSIS gene set. No genes overlapped among the three signatures, thereby avoiding correlations driven directly by shared gene components.

Associations of the M2BPGi‐related fibrosis and glycolysis‐related signatures with the mTORC1‐related anabolic signature were evaluated using Pearson correlation analysis. For survival analysis, patients were divided into high‐ and low‐signature groups according to the median mTORC1‐related anabolic signature score. OS was estimated using the Kaplan–Meier method and compared using the log‐rank test.

### 2.5 Statistical Analysis

For the Cox proportional hazards regression analyses, platelet count was dichotomized at 13 × 10^4^/μL according to a previously reported cutoff associated with impaired hepatic functional reserve in patients undergoing hepatectomy for HCC [16]. Continuous variables were presented as medians, and categorical variables as numbers and percentages. Continuous variables were compared using the Mann–Whitney *U* test. Categorical variables were compared using the *χ* ^2^ test or Fisher's exact test, as appropriate. OS and recurrence‐free survival (RFS) were estimated using the Kaplan–Meier method, and survival curves were compared using the log‐rank test. Cox regression was used to identify factors associated with OS and RFS. Variables with *p* < 0.05 in the univariate analyses were entered into the initial multivariable Cox models. Seven variables were initially entered into the RFS model and eight into the OS model. Backward elimination was subsequently performed to determine the final models. To assess the incremental prognostic value of M2BPGi and SUVmax, Harrell's C‐index was compared among the base models and models additionally incorporating M2BPGi, SUVmax, or both. The base models were constructed using conventional clinicopathological variables that were significant in the univariable analyses, excluding M2BPGi and SUVmax. Harrell's C‐index analyses were performed. All statistical tests were two‐sided, and *p* < 0.05 was considered statistically significant. Statistical analyses were performed using JMP Pro 17 (SAS Institute Inc., Cary, NC, USA), and Harrell's C‐index analyses were performed using R version 4.5.3 (R Foundation for Statistical Computing, Vienna, Austria).

## 3 Results

### 3.1 Study Cohort and Cutoff Values for M2BPGi and SUVmax

The study cohort comprised 291 patients with HCC who underwent hepatic resection and had available preoperative M2BPGi and ^18^F‐FDG PET/CT data. ROC curve analyses for OS identified optimal cutoff values of 1.38 for M2BPGi and 3.4 for SUVmax, with areas under the curve of 0.650 and 0.651, respectively (Figure S1). Based on these cutoff values, 181 patients were classified into the M2BPGi‐low group and 110 into the M2BPGi‐high group, whereas 108 patients were classified into the SUVmax‐low group and 183 into the SUVmax‐high group.

### 3.2 Clinicopathological Characteristics Associated With M2BPGi and SUVmax

Compared with the M2BPGi‐low group, the M2BPGi‐high group included a lower proportion of men (60.9% vs. 73.5%, *p* = 0.0249) and a higher proportion of patients with diabetes mellitus (48.1% vs. 34.5%, *p* = 0.0237) (Table 1). The M2BPGi‐high group had lower serum albumin (3.8 vs. 4.1 g/dL, *p* < 0.0001) and prothrombin time (89% vs. 98%, *p* < 0.0001), higher aspartate aminotransferase (35 vs. 28 U/L, *p* < 0.0001) and lactate dehydrogenase (196 vs. 186 U/L, *p* = 0.0284), and higher frequencies of modified albumin–bilirubin (mALBI) grade 2b (30.0% vs. 12.2%, *p* = 0.0002) and microscopic liver fibrosis graded F3 or F4 (46.4% vs. 26.5%, *p* = 0.0018). In contrast, M2BPGi status was not significantly associated with alpha‐fetoprotein (AFP), des‐*γ*‐carboxy prothrombin (DCP), tumor size, tumor number, poor differentiation, microscopic vascular invasion, microscopic intrahepatic metastasis, or SUVmax.

**TABLE 1:** Comparison of clinicopathological characteristics between the M2BPGi‐high group and low group.

	M2BPGi‐low (*N* = 181)	M2BPGi‐high (*N* = 110)	*p*
Age (years)	72 (65–78)	72 (67–78)	0.5388
Sex, male	133 (73.5)	67 (60.9)	0.0249
Diabetes mellitus	60 (34.5)	51 (48.1)	0.0237
HBs‐Ag, positive	32 (17.7)	18 (16.4)	0.7724
HCV‐Ab, positive	61 (34.1)	46 (42.6)	0.1484
Previous treatment for HCC, any	45 (24.9)	26 (23.6)	0.8134
Hepatic resection	44 (24.3)	25 (22.7)	0.7583
TACE/TAE	8 (4.4)	5 (4.6)	0.9599
Systemic therapy	8 (4.4)	8 (7.3)	0.3006
Total bilirubin (mg/dL)	0.8 (0.6–1.1)	0.8 (0.6–1.1)	0.7431
Albumin (g/dL)	4.1 (3.9–4.4)	3.8 (3.5–4.2)	< 0.0001
Prothrombin time (%)	98 (88–107)	89 (79–97)	< 0.0001
Platelet (×10^4^/μL)	18.5 (14.7–22.8)	13.9 (11.2–19.0)	< 0.0001
AST (U/L)	28 (22–36)	35 (24–54)	< 0.0001
ALT (U/L)	22 (17–33)	26 (16–44)	0.0657
LDH (U/L)	186 (159–209)	196 (167–222)	0.0284
Child‐Pugh, B	10 (5.5)	11 (10.0)	0.1526
mALBI, Grade 2b	22 (12.2)	33 (30.0)	0.0002
AFP (ng/mL)	6.8 (3.0–123.7)	6.4 (4.0–55.3)	0.6645
DCP (mAU/mL)	80 (27–694)	122 (28–1211)	0.5552
Tumor size (cm)	3.0 (1.8–4.9)	2.6 (2.0–4.4)	0.8051
Tumor number, multiple	29 (16.0)	24 (21.8)	0.4501
Poor differentiation, present	56 (30.9)	30 (27.3)	0.7854
Microscopic vascular invasion	33 (18.2)	21 (19.1)	0.8997
Microscopic intrahepatic metastasis	11 (6.1)	11 (10.1)	0.2086
Microscopic liver fibrosis, F3 or F4	48 (26.5)	51 (46.4)	0.0018
PET SUVmax	3.75 (3.01–5.00)	3.74 (3.04–5.72)	0.3517
M2BPGi	0.85 (0.65–1.1)	2.04 (1.60–3.15)	< 0.0001

*Note:* The data are presented as *n* (%) or median (IQR). Data on race and ethnicity were not collected because all participants were recruited from a single institution in Japan, where the population is ethnically homogeneous.

Abbreviations: AFP, alpha‐fetoprotein; ALT, alanine aminotransferase; AST, aspartate aminotransferase; DCP, des‐*γ*‐carboxy prothrombin; HBs‐Ag, hepatitis B surface antigen; HCV‐Ab, hepatitis C virus antibody; LDH, lactate dehydrogenase; mALBI, modified albumin–bilirubin; PET SUV, positron emission tomography standardized uptake value.

Stratification by SUVmax status showed a different pattern of clinicopathological associations (Table 2). Compared with the SUVmax‐low group, the SUVmax‐high group was slightly older (72 vs. 71 years, *p* = 0.0461) and had higher AFP (7.8 vs. 5.7 ng/mL, *p* = 0.0388) and DCP (210 vs. 36 mAU/mL, *p* < 0.0001), larger tumors (3.3 vs. 2.0 cm, *p* < 0.0001), and higher frequencies of poor differentiation (33.9% vs. 22.2%, *p* = 0.0137), microscopic vascular invasion (25.7% vs. 6.5%, *p* < 0.0001), and microscopic intrahepatic metastasis (11.6% vs. 1.9%, *p* = 0.0037). The overall frequency of previous treatment for HCC did not differ between the SUVmax groups, although systemic therapy was more frequent in the SUVmax‐high group (7.7% vs. 1.9%, *p* = 0.0360). Liver function and fibrosis variables (albumin, prothrombin time, mALBI grade, and microscopic liver fibrosis) and M2BPGi levels did not differ significantly according to SUVmax status. Thus, M2BPGi and SUVmax showed distinct clinicopathological association patterns and predominantly reflected background liver status and tumor aggressiveness, respectively.

**TABLE 2:** Comparison of clinicopathological characteristics between the SUVmax‐high group and low group.

	SUVmax‐low (*N* = 108)	SUVmax‐high (*N* = 183)	*p*
Age (years)	71 (64–77)	72 (67–78)	0.0461
Sex, male	81 (75.0)	119 (65.0)	0.0763
Diabetes mellitus	38 (36.2)	73 (41.7)	0.3603
HBs‐Ag, positive	24 (22.2)	26 (14.2)	0.0800
HCV‐Ab, positive	38 (35.5)	69 (37.7)	0.6329
Previous treatment for HCC, any	28 (25.9)	43 (23.5)	0.6412
Hepatic resection	28 (25.9)	41 (22.4)	0.4950
TACE/TAE	6 (5.6)	7 (3.8)	0.4900
Systemic therapy	2 (1.9)	14 (7.7)	0.0360
Total bilirubin (mg/dL)	0.8 (0.6–1.1)	0.8 (0.6–1.1)	0.8540
Albumin (g/dL)	4.1 (3.7–4.4)	4.0 (3.7–4.3)	0.0755
Prothrombin time (%)	94 (85–104)	95 (85–105)	0.9540
Platelet (×10^4^/μL)	16.2 (13.2–21.7)	17.1 (12.6–21.7)	0.6832
AST (U/L)	29 (22–39)	30 (23–42)	0.3668
ALT (U/L)	24 (16–38)	24 (17–36)	0.9571
LDH (U/L)	188 (163–210)	190 (160–219)	0.5131
Child‐Pugh, B	4 (3.7)	17 (9.3)	0.0752
mALBI, Grade 2b	16 (14.8)	39 (21.3)	0.1715
AFP (ng/mL)	5.7 (3.1–37.0)	7.8 (3.7–140.9)	0.0388
DCP (mAU/mL)	36 (23–108)	210 (36–1757)	< 0.0001
Tumor size (cm)	2.0 (1.3–3.2)	3.3 (2.4–5.4)	< 0.0001
Tumor number, multiple	14 (13.0)	39 (21.3)	0.1161
Poor differentiation, present	24 (22.2)	62 (33.9)	0.0137
Microscopic vascular invasion	7 (6.5)	47 (25.7)	< 0.0001
Microscopic intrahepatic metastasis	2 (1.9)	20 (11.6)	0.0037
Microscopic liver fibrosis, F3 or F4	42 (38.9)	57 (31.2)	0.0953
PET SUVmax	2.90 (2.80–3.10)	4.77 (3.84–6.33)	< 0.0001
M2BPGi	1.14 (0.73–1.78)	1.19 (0.78–1.67)	0.7505

*Note:* The data are presented as *n* (%) or median (IQR). Data on race and ethnicity were not collected because all participants were recruited from a single institution in Japan, where the population is ethnically homogeneous.

Abbreviations: AFP, alpha‐fetoprotein; ALT, alanine aminotransferase; AST, aspartate aminotransferase; DCP, des‐*γ*‐carboxy prothrombin; HBs‐Ag, hepatitis B surface antigen; HCV‐Ab, hepatitis C virus antibody; LDH, lactate dehydrogenase; mALBI, modified albumin‐bilirubin; PET SUV, positron emission tomography standardized uptake value.

### 3.3 Associations of M2BPGi and SUVmax With Postoperative Survival

**FIGURE 1:**
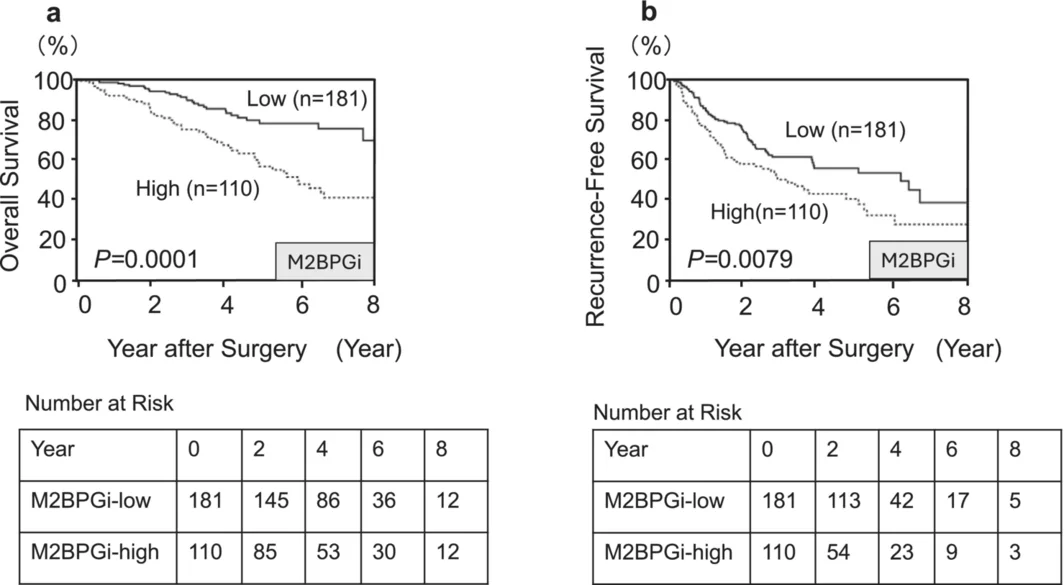
Overall and recurrence‐free survival according to M2BPGi status. Kaplan–Meier curves for (a) overall survival and (b) recurrence‐free survival according to preoperative M2BPGi status. Patients are classified as M2BPGi‐low or M2BPGi‐high using a cutoff value of 1.38. Survival curves are compared using the log‐rank test. M2BPGi, Mac‐2 binding protein glycosylation isomer.

**FIGURE 2:**
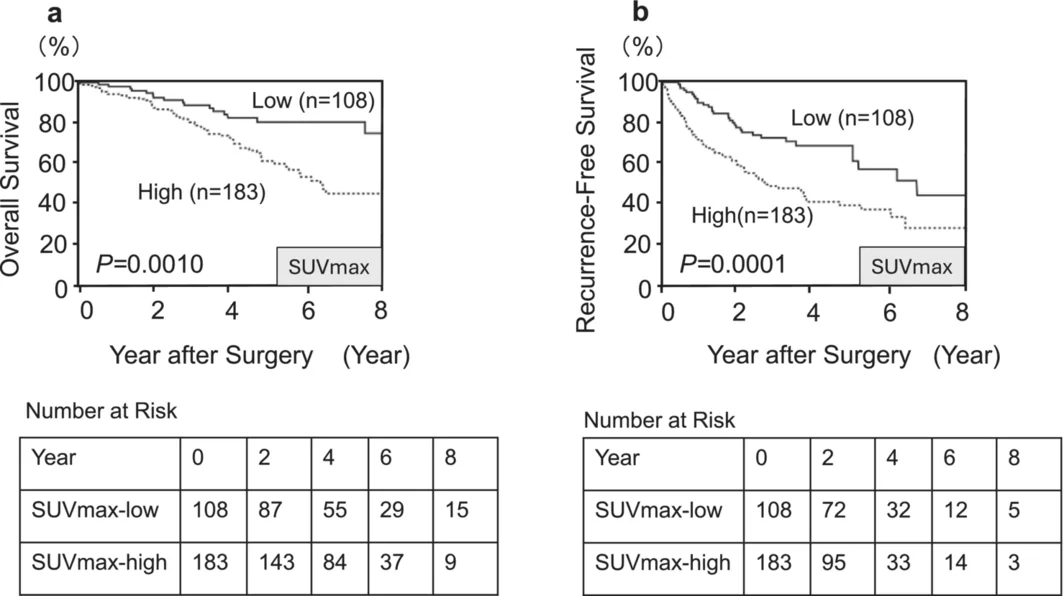
Overall and recurrence‐free survival according to SUVmax status. Kaplan–Meier curves for (a) overall survival and (b) recurrence‐free survival according to preoperative SUVmax status. Patients are classified as SUVmax‐low or SUVmax‐high using a cutoff value of 3.4. Survival curves are compared using the log‐rank test. SUVmax, maximum standardized uptake value.

Kaplan–Meier analysis showed that the M2BPGi‐high group had significantly worse OS (*p* = 0.0001; Figure 1a) and RFS (*p* = 0.0079; Figure 1b) than the M2BPGi‐low group. Similarly, the SUVmax‐high group had significantly worse OS (*p* = 0.0010; Figure 2a) and RFS (*p* = 0.0001; Figure 2b) than the SUVmax‐low group. Thus, each marker alone stratified postoperative recurrence and survival.

### 3.4 Independent Prognostic Factors for Recurrence‐Free and Overall Survival

During the follow‐up period, 125 cases experienced an RFS event, and 77 deaths were observed. In univariate analyses, mALBI grade 2b, Child‐Pugh class B, multiple tumors, microscopic vascular invasion, microscopic intrahepatic metastasis, M2BPGi‐high status, and SUVmax‐high status were significantly associated with worse RFS (Table 3). In multivariable analysis, multiple tumors (hazard ratio [HR], 2.07; 95% confidence interval [CI], 1.36–3.14; *p* = 0.0006), microscopic vascular invasion (HR, 1.73; 95% CI, 1.13–2.65; *p* = 0.0120), microscopic intrahepatic metastasis (HR, 2.55; 95% CI, 1.48–4.41; *p* = 0.0008), M2BPGi‐high status (HR, 1.61; 95% CI, 1.12–2.31; *p* = 0.0105), and SUVmax‐high status (HR, 1.94; 95% CI, 1.27–2.98; *p* = 0.0023) remained independently associated with worse RFS.

**TABLE 3:** Univariate and multivariate analyses of factors related to RFS in patients with HCC who had undergone hepatic resection (Cox proportional hazards analysis).

*N* = 291	Univariate analysis			Multivariate analysis		
	Hazard ratio	95% CI	*p*	Hazard ratio	95% CI	*p*
Age (years)	0.99	0.98–1.01	0.5413			
Sex: Male	1.12	0.76–1.65	0.5611			
Diabetes mellitus	1.09	0.76–1.57	0.6338			
HBs‐Ag positive	1.04	0.65–1.69	0.8611			
HCV‐Ab positive	1.32	0.92–1.90	0.1302			
Platelet, < 13 × 10^4^/μL	1.15	0.78–1.71	0.4859			
mALBI: Grade 2b (vs. Grade 1 or 2a)	1.56	1.03–2.37	0.0352	—	—	—
Child‐Pugh: B (vs. A)	2.11	1.19–3.77	0.0112	—	—	—
AFP (ng/mL)	1.00	1.00–1.00	0.5449			
DCP (mAU/mL)	1.00	1.00–1.00	0.0820			
Tumor size (cm)	1.07	0.99–1.13	0.0535			
Tumor number: multiple (vs. single)	2.45	1.64–3.65	< 0.0001	2.07	1.36–3.14	0.0006
Poor differentiation: present (vs. absent)	1.36	0.94–1.97	0.1060			
Microscopic vascular invasion: present (vs. absent)	2.36	1.58–3.52	< 0.0001	1.73	1.13–2.65	0.0120
Microscopic intrahepatic metastasis: present (vs. absent)	3.68	2.19–6.17	< 0.0001	2.55	1.48–4.41	0.0008
Microscopic liver fibrosis: F3 or F4 (vs. F0‐2)	1.27	0.89–1.84	0.4135			
M2BPGi High (vs. Low)	1.60	1.13–2.28	0.0085	1.61	1.12–2.31	0.0105
PET SUVmax High (vs. Low)	2.17	1.45–3.24	0.0002	1.94	1.27–2.98	0.0023

Abbreviations: AFP, alpha‐fetoprotein; BCLC staging, Barcelona Clinic Liver Cancer staging system; DCP, des‐*γ*‐carboxy prothrombin; HBs‐Ag, hepatitis B surface antigen; HCV‐Ab, hepatitis C virus antibody; mALBI, modified albumin‐bilirubin; SUVmax, Maximum Standardized Uptake Value; xCT, cystine/glutamate antiporter subunit.

The corresponding analyses for OS showed that mALBI grade 2b, Child‐Pugh class B, tumor size, multiple tumors, microscopic vascular invasion, microscopic intrahepatic metastasis, M2BPGi‐high status, and SUVmax‐high status were significantly associated with worse OS (Table 4). Multivariable analysis identified mALBI grade 2b (HR, 2.13; 95% CI, 1.30–3.51; *p* = 0.0029), microscopic intrahepatic metastasis (HR, 3.36; 95% CI, 1.82–6.21; *p* = 0.0001), M2BPGi‐high status (HR, 2.03; 95% CI, 1.25–3.28; *p* = 0.0042), and SUVmax‐high status (HR, 1.93; 95% CI, 1.11–3.37; *p* = 0.0199) as independent factors associated with worse OS.

**TABLE 4:** Univariate and multivariate analyses of factors related to OS in patients with HCC who had undergone hepatic resection (Cox proportional hazards analysis).

*N* = 291	Univariate analysis			Multivariate analysis		
	Hazard ratio	95% CI	*p*	Hazard ratio	95% CI	*p*
Age (years)	1.04	0.99–1.09	0.0755			
Sex: Male	0.76	0.47–1.21	0.2477			
Diabetes mellitus	0.95	0.60–1.51	0.8391			
HBs‐Ag positive	0.59	0.28–1.22	0.1539			
HCV‐Ab positive	1.10	0.69–1.74	0.6991			
Platelet, < 13 × 10^4^/μL	1.27	0.77–2.10	0.3469			
mALBI: Grade 2b (vs. Grade 1 or 2a)	2.83	1.77–4.53	< 0.0001	2.13	1.30–3.51	0.0029
Child‐Pugh: B (vs. A)	3.27	1.75–6.09	0.0011	—	—	—
AFP (ng/mL)	1.00	1.00–1.00	0.5547			
DCP (mAU/mL)	1.00	1.00–1.00	0.2748			
Tumor size (cm)	1.09	1.00–1.17	0.0392	—	—	—
Tumor number: multiple (vs. single)	1.81	1.09–3.02	0.0302	—	—	—
Poor differentiation: present (vs. absent)	1.50	0.95–2.38	0.0816			
Microscopic vascular invasion: present (vs. absent)	2.24	1.37–3.65	0.0024	—	—	—
Microscopic intrahepatic metastasis: present (vs. absent)	3.61	1.98–6.59	< 0.0001	3.36	1.82–6.21	0.0001
Microscopic liver fibrosis: F3 or F4 (vs. F0‐2)	1.04	0.64–1.67	0.8821			
M2BPGi High (vs. Low)	2.40	1.52–3.79	0.0002	2.03	1.25–3.28	0.0042
PET SUVmax High (vs. Low)	2.41	1.40–4.14	0.0015	1.93	1.11–3.37	0.0199

Abbreviations: AFP, alpha‐fetoprotein; BCLC staging, Barcelona Clinic Liver Cancer staging system; DCP, des‐*γ*‐carboxy prothrombin; HBs‐Ag, hepatitis B surface antigen; HCV‐Ab, hepatitis C virus antibody; mALBI, modified albumin‐bilirubin; SUVmax, Maximum Standardized Uptake Value; xCT, cystine/glutamate antiporter subunit.

### 3.5 Combined Prognostic Stratification Using M2BPGi and SUVmax

**FIGURE 3:**
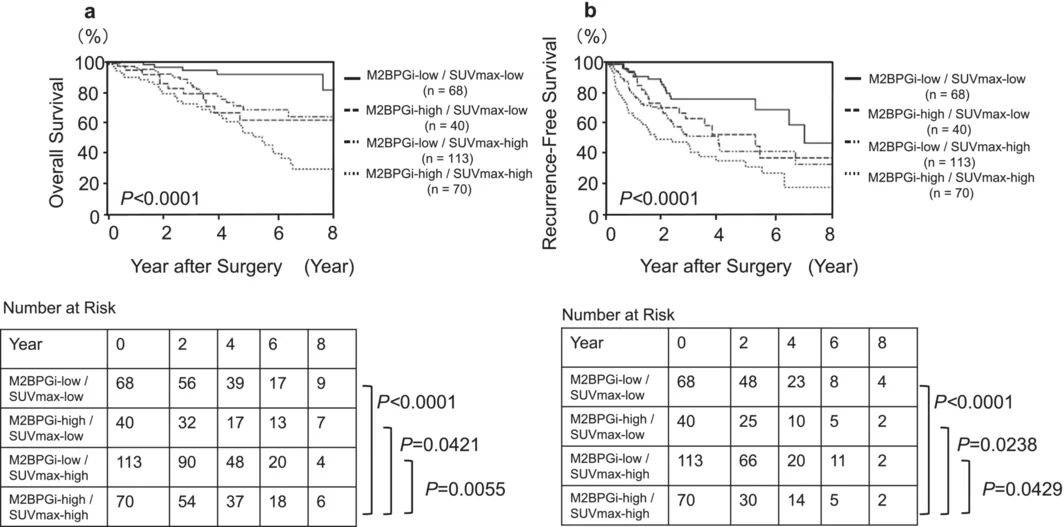
Prognostic stratification using the combination of M2BPGi and SUVmax. Kaplan–Meier curves for (a) overall survival and (b) recurrence‐free survival in the SUVmax‐low/M2BPGi‐low (*n* = 68), SUVmax‐low/M2BPGi‐high (*n* = 40), SUVmax‐high/M2BPGi‐low (*n* = 113), and SUVmax‐high/M2BPGi‐high (dual‐high; *n* = 70) groups. Overall differences among the four groups are assessed using the log‐rank test. Pairwise *p* values comparing the dual‐high group with each of the other three groups are shown below the graphs. M2BPGi, Mac‐2 binding protein glycosylation isomer; SUVmax, maximum standardized uptake value.

Patients were classified into four groups according to the combination of M2BPGi and SUVmax status: M2BPGi‐low/SUVmax‐low (*n* = 68), M2BPGi‐high/SUVmax‐low (*n* = 40), M2BPGi‐low/SUVmax‐high (*n* = 113), and M2BPGi‐high/SUVmax‐high (dual‐high; *n* = 70) (Figure 3a,b). OS and RFS differed significantly across the four groups (both *p* < 0.0001). The dual‐high group had significantly worse OS than the M2BPGi‐low/SUVmax‐low (*p* < 0.0001), M2BPGi‐high/SUVmax‐low (*p* = 0.0421), and M2BPGi‐low/SUVmax‐high groups (*p* = 0.0055). RFS showed the same pattern, with significantly worse outcomes in the dual‐high group than in the M2BPGi‐low/SUVmax‐low (*p* < 0.0001), M2BPGi‐high/SUVmax‐low (*p* = 0.0238), and M2BPGi‐low/SUVmax‐high groups (*p* = 0.0429). Thus, the dual‐high classification identified the subgroup at the highest risk of recurrence and death after hepatic resection. The addition of both M2BPGi and SUVmax to the base models increased Harrell's C‐index from 0.65 to 0.69 for RFS and from 0.70 to 0.73 for OS (Table S2).

To further characterize this high‐risk subgroup, its clinicopathological features were compared with those of the other three groups (Table S1). The dual‐high group had lower albumin (3.8 vs. 4.1 g/dL, *p* < 0.0001) and prothrombin time (88% vs. 95%, *p* = 0.0008), higher frequencies of Child‐Pugh class B (12.9% vs. 5.4%, *p* = 0.0364) and mALBI grade 2b (32.9% vs. 14.5%, *p* = 0.0006), higher DCP levels (293 vs. 68 mAU/mL, *p* = 0.0022), larger tumors (3.5 vs. 2.7 cm, *p* = 0.0051), and higher frequencies of microscopic vascular invasion (30.0% vs. 14.9%, *p* = 0.0047) and microscopic intrahepatic metastasis (16.7% vs. 5.2%, *p* = 0.0027).

### 3.6 Subgroup Analysis of the Combined Classification According to mALBI Grade

Given the independent association between mALBI grade and OS, exploratory subgroup analyses were performed to determine whether the combined M2BPGi–SUVmax classification retained prognostic value after stratification by liver function (Figure S2). Among the 236 patients with mALBI Grade 1 or 2a, OS differed significantly across the four combined groups (*p* = 0.0012; Figure S2a). The dual‐high group had significantly worse OS than the M2BPGi‐low/SUVmax‐low group (*p* = 0.0001) and the M2BPGi‐low/SUVmax‐high group (*p* = 0.0129). The difference from the M2BPGi‐high/SUVmax‐low group showed a similar trend but was not statistically significant (*p* = 0.0933). Among the 55 patients with mALBI grade 2b, OS did not differ significantly across the four groups (*p* = 0.2846; Figure S2b). These exploratory findings suggest that the combined M2BPGi–SUVmax classification remained prognostically informative even among patients with preserved liver function. Compared with patients with mALBI Grade 1 or 2a, those with mALBI grade 2b had poorer liver function, a higher frequency of F3–F4 fibrosis, and higher M2BPGi levels, whereas SUVmax and tumor‐related characteristics did not differ significantly (Table S3).

### 3.7 Associations of Fibrosis‐ and Glycolysis‐Related Signatures With mTORC1‐Related Activity

We performed an exploratory transcriptomic analysis of the publicly available TCGA‐LIHC dataset in GEPIA3. Both the M2BPGi‐related fibrosis signature and the glycolysis‐related signature were positively correlated with the mTORC1‐related anabolic signature (*R* = 0.439 and *R* = 0.670, respectively; *p* < 0.0001; Figure S3a,b). A high mTORC1‐related anabolic signature was also associated with worse OS (*p* = 0.0074; Figure S3c).

## 4 Discussion

In this study, preoperative M2BPGi identified patients with HCC who had more advanced liver fibrosis and impaired hepatic reserve, and it independently predicted recurrence and death after hepatic resection. Adding tumor metabolic activity further identified a subgroup with both an unfavorable background liver and aggressive tumor biology.

M2BPGi is a serum glycan biomarker of hepatic fibrosis. High M2BPGi was associated with lower albumin and prothrombin time values and with higher frequencies of mALBI grade 2b and F3–F4 fibrosis. In contrast, M2BPGi was not associated with tumor size, tumor number, differentiation, vascular invasion, or SUVmax. These findings suggest that M2BPGi primarily reflects fibrosis and functional impairment of the underlying liver rather than tumor‐related features. Previous studies have shown that preoperative M2BPGi is associated with fibrosis in the non‐tumorous liver and predicts recurrence and survival after HCC resection [17]. Other reports have demonstrated that M2BPGi predicts early postoperative recurrence, correlates with fibrosis markers and the albumin–bilirubin (ALBI) score, and independently predicts both RFS and OS [6, 7, 18]. Our findings are consistent with these observations.

Because a fibrosis‐related marker alone cannot fully capture tumor behavior, we assessed tumor metabolic activity as a complementary factor. High tumor metabolic activity was associated with higher AFP and DCP levels, larger tumors, poor differentiation, microscopic vascular invasion, and microscopic intrahepatic metastasis, but not with liver function, microscopic fibrosis, or M2BPGi. Previous studies have similarly linked high ^18^F‐FDG uptake to microscopic vascular invasion, early recurrence, and poor survival after hepatic resection for HCC [8, 19]. Recent studies have also linked metabolic and redox adaptations to ferroptosis resistance and unfavorable outcomes in HCC [20, 21]. Therefore, tumor metabolic activity may provide information on tumor aggressiveness that is distinct from the background liver status reflected by M2BPGi.

M2BPGi and SUVmax were not significantly correlated and were associated with different clinicopathological features. The dual‐high group had poorer liver function and more aggressive tumor characteristics, including higher DCP levels, larger tumors, microscopic vascular invasion, and intrahepatic metastasis. Additionally, this group had the poorest RFS and OS. These findings suggest that combining M2BPGi and SUVmax may identify patients with both an unfavorable background liver and aggressive tumor biology. Recurrence after HCC resection is biologically heterogeneous. Recurrence driven by aggressive tumor biology is generally considered to reflect intrahepatic metastasis from the primary tumor and is often associated with earlier recurrence, whereas recurrence arising from the chronically diseased liver may reflect multicentric or de novo hepatocarcinogenesis and tends to occur later [22]. In this context, SUVmax may predominantly reflect tumor‐related recurrence risk, whereas M2BPGi may capture the carcinogenic potential of the underlying liver. Thus, their combination may be clinically meaningful by integrating two biologically distinct pathways of HCC recurrence.

The mTORC1 pathway may partly explain this association. Previous studies have shown that M2BPGi can promote HCC cell proliferation and invasion through galectin‐3‐mediated activation of mTORC1 signaling [23]. Radiogenomic analysis has also shown that ^18^F‐FDG‐high HCCs exhibit increased mTORC1‐related signaling and poorer postoperative RFS [24]. mTORC1 activation has also been implicated in non‐alcoholic fatty liver disease‐associated hepatocarcinogenesis, with long non‐coding RNA SNHG6‐mediated cholesterol sensing promoting mTORC1 activation and progression from non‐alcoholic fatty liver disease to HCC [25]. Consistent with these observations, the M2BPGi‐related fibrosis signature and the glycolysis‐related signature were both positively correlated with an mTORC1‐related anabolic signature in TCGA‐LIHC. Moreover, a high mTORC1‐related signature was associated with worse OS. These results suggest that fibrosis‐related stromal activity and enhanced glycolytic metabolism, although clinically distinct, may partly converge on mTORC1‐related anabolic signaling. However, these transcriptomic associations do not demonstrate that M2BPGi directly activates mTORC1 or that mTORC1 mediates the poor prognosis of the dual‐high group. The analysis was based on bulk tumor RNA expression and did not assess protein phosphorylation or cellular localization.

In the exploratory subgroup analysis by mALBI grade, the combined classification significantly stratified OS even among patients with mALBI Grade 1 or 2a. M2BPGi may therefore provide information on hepatic fibrosis and on the biological environment of the underlying liver that albumin and bilirubin do not fully capture [26]. Patients with mALBI grade 2b had more advanced fibrosis and higher M2BPGi levels, whereas SUVmax did not differ according to mALBI grade. This overlap between impaired liver function and elevated M2BPGi may partly explain the reduced prognostic discrimination of the combined classification in the mALBI grade 2b subgroup. However, the small sample size of this subgroup limits definitive interpretation. Larger studies are needed to determine whether the classification is also useful in patients with more impaired liver function.

Beyond long‐term oncologic outcomes, assessment of the background liver is also important for perioperative outcomes after hepatectomy. Liver function grade has been associated with post‐hepatectomy liver failure [27], and the preoperative FIB‐4 index has been reported to be associated with severe postoperative complications and organ failure [28]. Since M2BPGi reflects liver fibrosis and hepatic function, it may also be useful for assessing perioperative risk. Thus, the combination of M2BPGi and SUVmax may provide information on both perioperative risk and long‐term oncologic outcomes. However, PHLF and postoperative complications were not evaluated in this study, and further studies are needed to determine its clinical value for predicting perioperative outcomes.

Both M2BPGi and tumor metabolic activity can be assessed preoperatively, allowing risk stratification without waiting for pathological findings. The combined use of M2BPGi and SUVmax also improved prognostic discrimination beyond conventional clinicopathological factors for both RFS and OS. Patients in the dual‐high group may warrant particularly careful postoperative surveillance. However, this study did not evaluate the effects of surveillance or therapeutic interventions based on this classification. External validation is therefore required before the classification can be used to guide follow‐up or treatment decisions.

This study has several limitations. First, it was a retrospective, single‐center study restricted to patients with both preoperative M2BPGi measurements and ^18^F‐FDG PET/CT, which may have introduced selection bias. Second, the cohort included primary resections, resections in previously treated patients, and repeat resections, resulting in clinical heterogeneity. Third, the M2BPGi and SUVmax cutoff values were derived using conventional ROC analyses and evaluated in the same cohort, which may have resulted in optimistic estimates of prognostic performance. Therefore, these cutoff values should be considered exploratory and require validation in an independent cohort. Fourth, the multivariable models were constructed using univariate screening followed by data‐driven variable reduction, which may have resulted in model instability and overfitting. External validation is therefore required. Fifth, the public database analysis was exploratory and used bulk transcriptomic data from tumor tissue. The M2BPGi‐related fibrosis signature cannot directly reproduce serum M2BPGi levels or fibrosis in the non‐tumorous liver, and the mTORC1‐related signature does not directly measure mTORC1 kinase activity. Finally, the mALBI‐stratified analyses were exploratory and included limited numbers of patients.

In conclusion, preoperative M2BPGi and SUVmax were independent prognostic markers that reflected the underlying liver condition and tumor aggressiveness, respectively, in patients with HCC undergoing hepatic resection. Their combination identified a dual‐high group with the highest risks of recurrence and death. This simple preoperative classification may be useful for risk stratification.

## Author Contributions

**Takeo Toshima:** data curation. **Junya Mita:** conceptualization, methodology, data curation, software, formal analysis, writing – original draft, writing – review and editing. **Yoshiyuki Kitamura:** data curation. **Tomoharu Yoshizumi:** writing – review and editing, supervision, funding acquisition. **Kojiro Shirabe:** conceptualization, methodology, data curation, software, formal analysis, writing – original draft, writing – review and editing. **Shinji Itoh:** conceptualization, methodology, data curation, software, formal analysis, writing – original draft, writing – review and editing, funding acquisition. **Norifumi Iseda:** data curation. **Takuro Isoda:** data curation. **Kousei Ishigami:** writing – review and editing.

## Funding

This study was supported by the Japan Society for the Promotion of Science KAKENHI (grant numbers JP‐26K02294 and JP‐26K19605).

## Ethics Statement

This study was approved by the Ethics Committee of Kyushu University (approval code: 23339).

## Consent

Opt‐out consent was obtained.

## Conflicts of Interest

Dr. Tomoharu Yoshizumi is an Editorial Board Member of *Annals of Gastroenterological Surgery*.

## Supporting information


**Figure S1:** Receiver operating characteristic curves for (a) M2BPGi and (b) SUVmax based on vital status during follow‐up. The optimal cutoff values were determined by maximizing the Youden index. The cutoff values are 1.38 for M2BPGi and 3.4 for SUVmax, with areas under the curve of 0.650 and 0.651, respectively.
**Figure S2:** Subgroup analysis of the combined classification according to mALBI grade. Kaplan–Meier curves for overall survival according to the combined M2BPGi–SUVmax classification among patients with (a) mALBI Grade 1 or 2a (*n* = 236) and (b) mALBI grade 2b (*n* = 55). Overall differences among the four groups are assessed using the log‐rank test. Pairwise *p* values comparing the dual‐high group with each of the other three groups are shown below the graphs.
**Figure S3:** Transcriptomic associations of fibrosis‐ and glycolysis‐related signatures with mTORC1‐related anabolic activity in TCGA‐LIHC. (a) Pearson correlation between the M2BPGi‐related fibrosis signature and the mTORC1‐related anabolic signature. (b) Pearson correlation between the glycolysis‐related signature and the mTORC1‐related anabolic signature. (c) Kaplan–Meier analysis of overall survival according to the mTORC1‐related anabolic signature. Patients are divided into high‐ and low‐signature groups using the median score; survival curves are compared using the log‐rank test. The mTORC1‐related anabolic, M2BPGi‐related fibrosis, and glycolysis‐related signatures comprise ASNS/MTHFD2/PSAT1/SLC7A5/RRM2, LGALS3BP/ACTA2/COL1A1/COL3A1, and SLC2A1/HK2/PFKP/PKM/LDHA, respectively.

**Table S1:** Comparison of clinicopathological characteristics between the SUVmax‐high/M2BPGi‐high group and the other groups.
**Table S2:** Incremental prognostic value of M2BPGi and SUVmax assessed by Harrell's C‐index.
**Table S3:** Comparison of clinicopathological characteristics between the mALBI Grade 1 or 2a group and mALBI Grade 2b group.

## Data Availability

The data that support the findings of this study are available on request from the corresponding author. The data are not publicly available due to privacy or ethical restrictions.
