# Supplementary material for: Mac‐2 Binding Protein Glycosylation Isomer (M2BPGi)–Based Risk Stratification Refined by Tumor Metabolic Activity in Hepatocellular Carcinoma
Source: Ann Gastroenterol Surg. 2026 Sep 1:10.1002/ags3.70272. Online ahead of print. doi: 10.1002/ags3.70272 (PMC13531093; doi:10.1002/ags3.70272)
Supplement: Supplementary file 1 [file AGS3-9999-0-s002.pdf]

Figure S1.

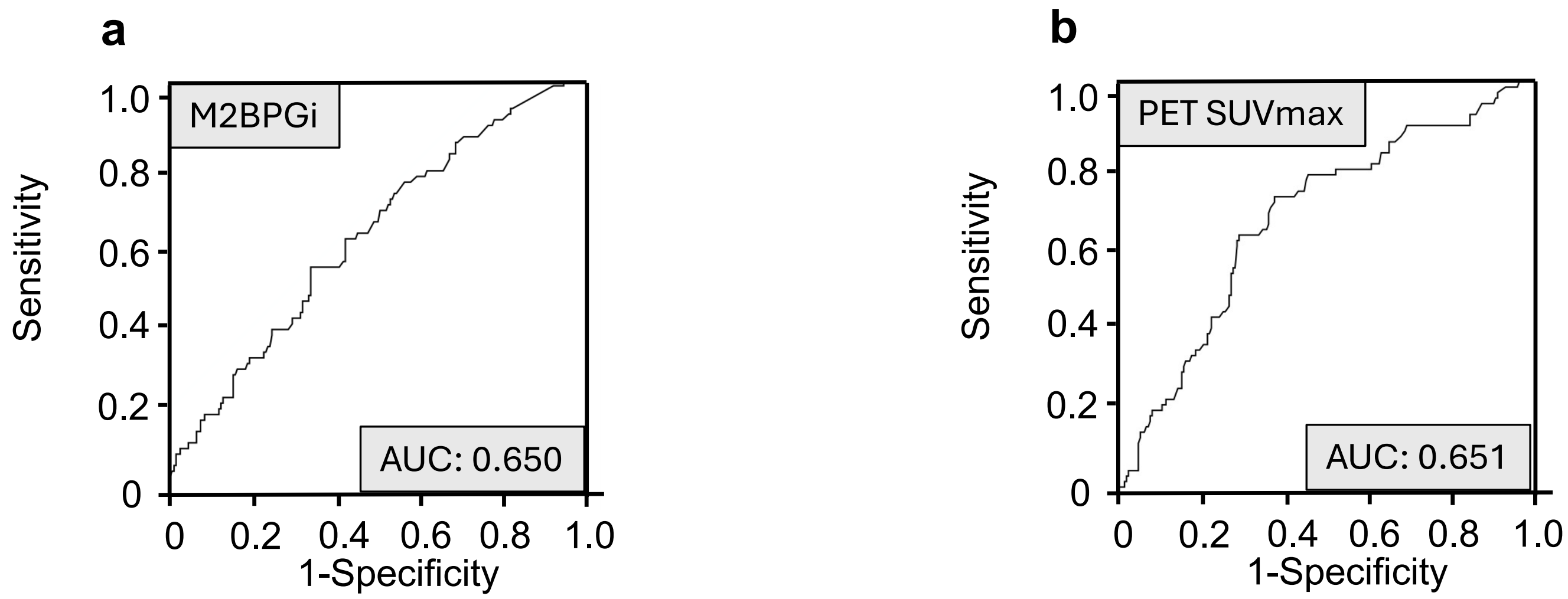

Figure S1. Receiver operating characteristic curves for (a) M2BPGi and (b) SUVmax based on vital status during follow-up. The optimal cutoff values were determined by maximizing the Youden index. The cutoff values are 1.38 for M2BPGi and 3.4 for SUVmax, with areas under the curve of 0.650 and 0.651, respectively.

M2BPGi, Mac-2 binding protein glycosylation isomer; SUVmax, maximum standardized uptake value; AUC, area under the curve.

Figure S2.

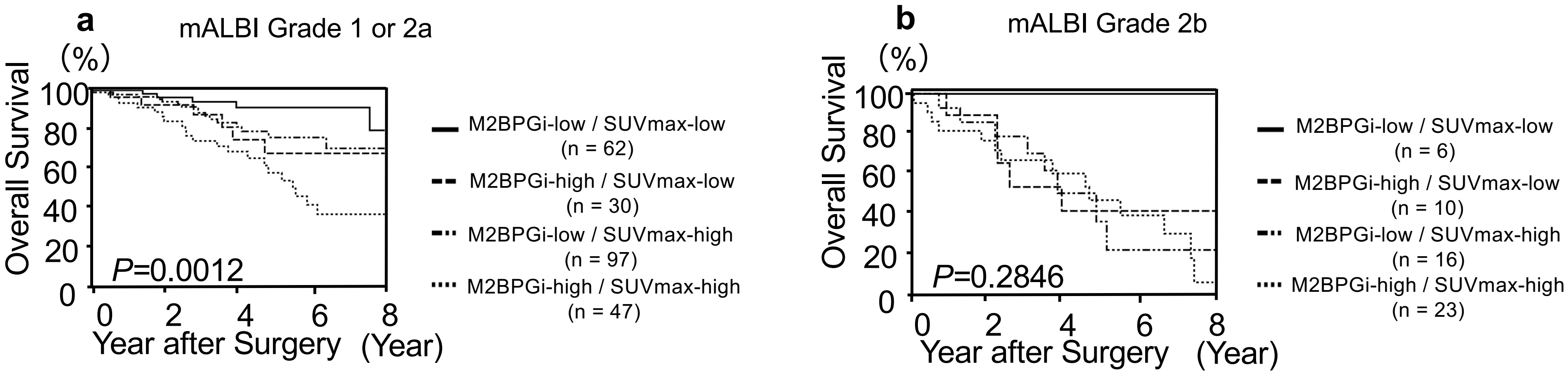

Number at Risk

Year	0	2	4	6	8
M2BPGi-low / SUVmax-low	62	52	36	16	8
M2BPGi-high / SUVmax-low	30	24	13	10	5
M2BPGi-low / SUVmax-high	97	78	44	18	4
M2BPGi-high / SUVmax-high	47	38	27	13	6

P=0.0001
P=0.0933
P=0.0129

Number at Risk

Year	0	2	4	6	8
M2BPGi-low / SUVmax-low	6	1	1	1	1
M2BPGi-high / SUVmax-low	10	9	5	4	3
M2BPGi-low / SUVmax-high	16	13	5	3	1
M2BPGi-high / SUVmax-high	23	17	11	6	1

P=0.0511
P=0.5380
P=0.9150

Figure S2. Subgroup analysis of the combined classification according to mALBI grade.

Kaplan–Meier curves for overall survival according to the combined M2BPGi–SUVmax classification among patients with (a) mALBI grade 1 or 2a (n = 236) and (b) mALBI grade 2b (n = 55). Overall differences among the four groups are assessed using the log-rank test. Pairwise P values comparing the dual-high group with each of the other three groups are shown below the graphs.

M2BPGi, Mac-2 binding protein glycosylation isomer; SUVmax, maximum standardized uptake value; mALBI, modified albumin–bilirubin.

Figure S3.

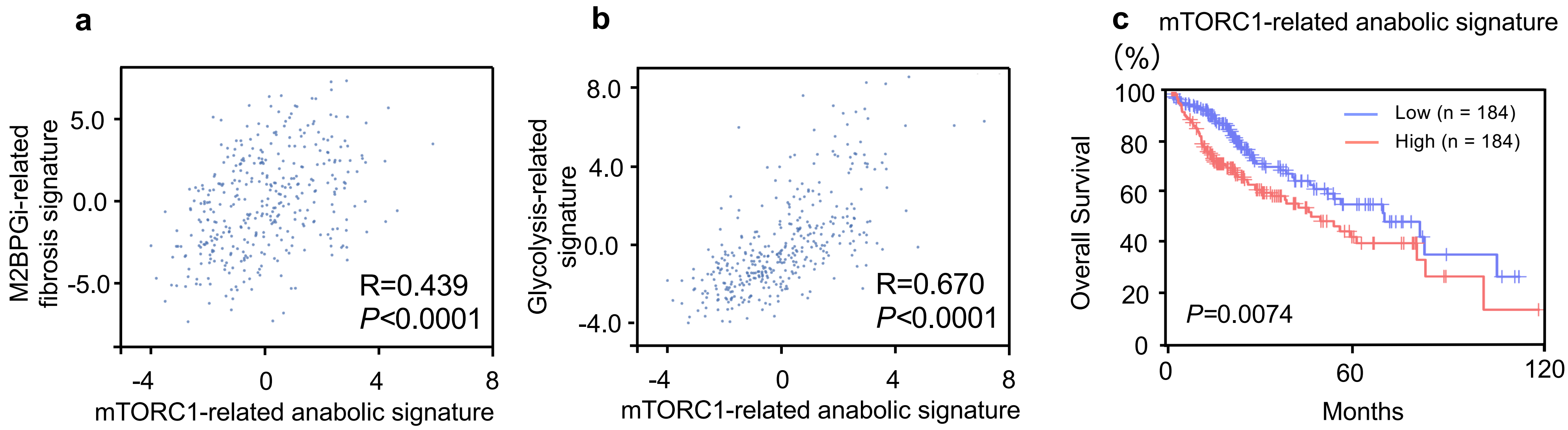

Figure S3. Transcriptomic associations of fibrosis- and glycolysis-related signatures with mTORC1-related anabolic activity in TCGA-LIHC. (a) Pearson correlation between the M2BPGi-related fibrosis signature and the mTORC1-related anabolic signature. (b) Pearson correlation between the glycolysis-related signature and the mTORC1-related anabolic signature. (c) Kaplan–Meier analysis of overall survival according to the mTORC1-related anabolic signature. Patients are divided into high- and low-signature groups using the median score; survival curves are compared using the log-rank test. The mTORC1-related anabolic, M2BPGi-related fibrosis, and glycolysis-related signatures comprise ASNS/MTHFD2/PSAT1/SLC7A5/RRM2, LGALS3BP/ACTA2/COL1A1/COL3A1, and SLC2A1/HK2/PFKP/PKM/LDHA, respectively.

TCGA-LIHC, The Cancer Genome Atlas liver hepatocellular carcinoma cohort; M2BPGi, Mac-2 binding protein glycosylation isomer; mTORC1, mechanistic target of rapamycin complex 1.
