# Supplementary material for: Mac‐2 Binding Protein Glycosylation Isomer (M2BPGi)–Based Risk Stratification Refined by Tumor Metabolic Activity in Hepatocellular Carcinoma
Source: Ann Gastroenterol Surg. 2026 Sep 1:10.1002/ags3.70272. Online ahead of print. doi: 10.1002/ags3.70272 (PMC13531093; doi:10.1002/ags3.70272)
Supplement: Supplementary file 2 [file AGS3-9999-0-s001.docx]

**Table S1.  Comparison of clinicopathological characteristics between the SUVmax-high/M2BPGi-high group and the other groups**

|  | M2BPGi-high/  SUVmax-high  (N = 70) | Others  (N = 221) | p-value |
| --- | --- | --- | --- |
| Age (years) | 72 (67 – 78) | 72 (67 – 78) | 0.4700 |
| Sex, male | 38 (54.3) | 162 (73.3) | 0.0028 |
| Diabetes mellitus | 33 (49.3) | 78 (36.6) | 0.0652 |
| HBs-Ag, positive | 9 (12.9) | 41 (18.6) | 0.2710 |
| HCV-Ab, positive  Previous treatment for HCC, any  Hepatic resection  TACE/TAE  Systemic therapy | 31 (45.6)  17 (24.3)  16 (22.9)  4 (5.7)  7 (10.0) | 76 (34.7)  54 (24.4)  53 (24.0)  9 (4.1)  9 (4.1) | 0.1049  0.9799  0.8471  0.5623  0.0580 |
| Total bilirubin (mg/dL)  Albumin (g/dL)  Prothrombin time (%)  Platelet (x10^4^/μL)  AST (U/L)  ALT (U/L) | 0.8 (0.6 – 1.0)  3.8 (3.5 – 4.1)  88 (79 – 100)  11.4 (8.5 – 14.2)  34 (22 – 52)  24 (16 – 40) | 0.8 (0.6 – 1.1)  4.1 (3.8 – 4.3)  95 (87 – 105)  14.2 (11.4 – 19.0)  29 (23 – 38)  24 (17 – 36) | 0.3926  < 0.0001  0.0008  0.0021  0.0631  0.9775 |
| LDH (U/L) | 197 (167 – 220) | 187 (161 – 214) | 0.1115 |
| Child-Pugh, B  mALBI, Grade 2b  AFP (ng/mL)  DCP (mAU/mL)  Tumor size (cm)  Tumor number, multiple  Poor differentiation, present  Microscopic vascular invasion  Microscopic intrahepatic metastasis  Microscopic liver fibrosis, F3 or F4  M2BPGi  PET SUVmax | 9 (12.9)  23 (32.9)  9.1 (4.5 – 295.9)  293 (37 – 4340)  3.5 (2.4 – 5.3)  17 (24.3)  23 (32.9)  21 (30.0)  11 (16.7)  30 (42.9)  1.94 (1.57 – 2.91)  5.09 (3.83 – 7.16) | 12 (5.4)  32 (14.5)  5.9 (3.1 – 76.4)  68 (26 – 527)  2.7 (1.7 – 4.2)  36 (16.3)  63 (28.5)  33 (14.9)  11 (5.2)  69 (31.2)  0.96 (0.70 – 1.29)  3.40 (2.90 – 4.73) | 0.0364  0.0006  0.0649  0.0022  0.0051  0.1309  0.4869  0.0047  0.0027  0.0911  < 0.0001  < 0.0001 |

The data are presented as n (%) or median (IQR).

Abbreviations; HBs-Ag: hepatitis B surface antigen, HCV-Ab: hepatitis C virus antibody, AST: aspartate aminotransferase, ALT: alanine aminotransferase, LDH: lactate dehydrogenase, mALBI: modified albumin-bilirubin, AFP: alpha-fetoprotein, DCP: des-γ-carboxy prothrombin, PET SUV: positron emission tomography standardized uptake value.

**Table S2.  Incremental prognostic value of M2BPGi and SUVmax assessed by Harrell’s C-index**

|  | Model | Harrell’s C-index | 95% CI |
| --- | --- | --- | --- |
| RFS | Base Model  Base Model + SUVmax  Base Model + M2BPGi  Base Model + SUVmax + M2BPGi | 0.65  0.68  0.67  0.69 | 0.59 – 0.70  0.63 – 0.74  0.61 – 0.72  0.64 – 0.74 |
| OS | Base Model  Base Model + SUVmax  Base Model + M2BPGi  Base Model + SUVmax + M2BPGi | 0.70  0.71  0.72  0.73 | 0.64 – 0.76  0.64 – 0.78  0.65 – 0.78  0.66 – 0.79 |

The base models were constructed using the conventional clinicopathological variables that were significant in the univariable analyses shown in Tables 3 and 4, excluding M2BPGi and SUVmax. The RFS base model included mALBI grade, Child–Pugh class, tumor number, microscopic vascular invasion, and microscopic intrahepatic metastasis. The OS base model included mALBI grade, Child–Pugh class, tumor size, tumor number, microscopic vascular invasion, and microscopic intrahepatic metastasis.

Abbreviations; M2BPGi, Mac-2 binding protein glycosylation isomer; mALBI, modified albumin–bilirubin; OS, overall survival; RFS, recurrence-free survival; SUVmax, maximum standardized uptake value.

**Table S3.  Comparison of clinicopathological characteristics between the mALBI Grade 1 or 2a group and mALBI Grade 2b group**

|  | mALBI Grade 1 or 2a  (N = 236) | mALBI Grade2b  (N = 55) | p-value |
| --- | --- | --- | --- |
| Age (years) | 72 (65 – 78) | 73 (67 – 78) | 0.3426 |
| Sex, male | 167 (70.8) | 33 (60.0) | 0.1210 |
| Diabetes mellitus | 84 (35.6) | 27 (49.1) | 0.0618 |
| HBs-Ag, positive | 44 (18.6) | 6 (10.9) | 0.1709 |
| HCV-Ab, positive  Previous treatment for HCC, any  Hepatic resection  TACE/TAE  Systemic therapy | 85 (36.0)  57 (24.2)  55 (23.3)  12 (5.1)  11 (4.7) | 22 (40.0)  14 (25.5)  14 (25.5)  1 (1.8)  5 (9.1) | 0.6429  0.8396  0.7357  0.2909  0.1943 |
| Total bilirubin (mg/dL)  Albumin (g/dL)  Prothrombin time (%)  Platelet (x10^4^/μL)  AST (U/L)  ALT (U/L) | 0.8 (0.6 – 1.1)  4.1 (3.9 – 4.4)  96 (88 – 105)  16.9 (13.4 – 21.2)  28 (22 – 39)  24 (17 – 38) | 1.0 (0.7 – 1.2)  3.4 (3.2 – 3.5)  80 (74 – 88)  15.7(10.2 – 23.1)  33 (23 – 47)  23 (16 – 35) | 0.0029  < 0.0001  < 0.0001  0.2829  0.1517  0.2994 |
| LDH (U/L) | 188 (160 – 214) | 195 (167 – 241) | 0.1548 |
| Child-Pugh, B  AFP (ng/mL)  DCP (mAU/mL)  Tumor size (cm)  Tumor number, multiple  Poor differentiation, present  Microscopic vascular invasion  Microscopic intrahepatic metastasis  Microscopic liver fibrosis, F3 or F4  M2BPGi  PET SUVmax | 3 (1.3)  6.6 (3.2 – 126.8)  78 (27 – 790)  3.0 (1.8 – 4.5)  45 (19.1)  72 (30.5)  45 (19.1)  16 (6.8)  72 (30.5)  1.10 (0.72 – 1.57)  3.72 (3.00 – 5.23) | 18 (32.7)  6.6 (4.4 – 55.1)  152 (26 – 1692)  2.6 (2.0 – 5.3)  8 (14.6)  14 (25.5)  9 (16.4)  6 (10.9)  27 (49.1)  1.55 (0.97 – 2.90)  4.21 (3.19 – 5.40) | < 0.0001  0.8421  0.2728  0.5340  0.4339  0.4594  0.6422  0.2873  0.0088  < 0.0001  0.1920 |

The data are presented as n (%) or median (IQR).

Abbreviations; HBs-Ag: hepatitis B surface antigen, HCV-Ab: hepatitis C virus antibody, AST: aspartate aminotransferase, ALT: alanine aminotransferase, LDH: lactate dehydrogenase, mALBI: modified albumin-bilirubin, AFP: alpha-fetoprotein, DCP: des-γ-carboxy prothrombin, PET SUV: positron emission tomography standardized uptake value.
